# The Effect of Nut Consumption on Diet Quality, Cardiometabolic and Gastrointestinal Health in Children: A Systematic Review of Randomized Controlled Trials

**DOI:** 10.3390/ijerph18020454

**Published:** 2021-01-08

**Authors:** Lauren C Mead, Alison M Hill, Sharayah Carter, Alison M Coates

**Affiliations:** 1 UniSA Allied Health and Human Performance, University of South Australia, Adelaide 5000, Australia; lauren.mead@mymail.unisa.edu.au (L.C.M.); sharayah.carter@unisa.edu.au (S.C.); 2 Alliance for Research in Exercise, Nutrition and Activity (ARENA), University of South Adelaide, Adelaide 5000, Australia; alison.hill@unisa.edu.au; 3 UniSA Clinical & Health Sciences, University of South Australia, Adelaide 5000, Australia

**Keywords:** tree nuts, child, diet quality, cardiometabolic health, gastrointestinal health

## Abstract

Tree nuts and ground nuts are nutrient-rich foods known to improve human health when consumed regularly in the diet. Past observational studies suggest that nuts improve adult and child health; however, limited randomized control trials (RCTs) have assessed the health effects of nuts in children. Using a systematic review approach, we examined the effect of nut intake on health outcomes in children aged 8–18 years. We searched PubMed, Scopus, Web of Science, EMBASE and Cochrane library to identify RCTs of interest. A total of 5783 articles were identified, 4821 were screened by title and abstract and 37 by full text resulting in four articles that met the inclusion criteria for the review. Nut consumption was between 15 and 30 g with durations of between 3 and 16 weeks. Nut consumption was shown to improve children’s diet quality (increase children’s intake of essential nutrients including fats (monounsaturated and polyunsaturated fats), protein and fiber), there were inconsistent effects on biomarkers of cardiometabolic health (improve lipid profiles, microvascular reactivity and inflammation) and gastrointestinal health (increase in the proportion of beneficial fecal bacteria). Further studies exploring the broad health benefits of nuts in children are needed with consideration given to higher doses and longer intervention periods.

## 1. Introduction

Nuts, including ground nuts and tree nuts such as almonds, cashews, pine nuts, walnuts, Brazil nuts, pistachio nuts and macadamia nuts, are a globally consumed snack food [1,2,3]. They contain numerous health-promoting nutrients and a suite of bioactive non-nutritive components; they are high in protein and unsaturated fats, fiber, plant sterols, minerals (calcium, potassium, magnesium), vitamins (B groups vitamins and vitamin E) and polyphenol antioxidants [4,5]. The inclusion of nuts in the diet has broad health benefits [6,7,8] and has been shown to improve overall diet quality and increase nutrient intake in several population surveys [3,9,10,11,12,13]. Data from the National Health and Nutrition Examination Survey (NHANES) showed that adults (>29 years or older) who consumed ~7 g/day tree nuts or the equivalent in tree nut butter had a higher diet quality score and better nutrient intake compared to adults who did not consume tree nuts or tree nut butter [14]. Specifically, tree nut or tree nut butter consumption was associated with increased intake of fiber, vitamin E, calcium and reduced sodium intake [14].

Observational and intervention studies suggest that when regularly consumed, tree nuts and peanuts reduce the risk of developing chronic diseases and associated risk factors [6,7,8]. Adults who frequently consume nuts (on average 28 g one or more times a week) have a reduced risk of developing cardiovascular disease (CVD), diabetes and have a lower incidence of metabolic syndrome [6,15,16,17]. These benefits may result from improvements in several established and emerging risk factors for CVD and diabetes following nut consumption, including blood pressure, lipids and lipoproteins, adiposity, glucose and insulin metabolism [16,18,19,20]. Nuts have also been suggested to have a prebiotic effect on the gut microbiota with mixed evidence of changes to the diversity and functionality of the gut [21]. The fiber and monounsaturated fat content of nuts are proposed to increase microbial diversity and reduced low-grade systemic inflammation [22,23].

In comparison to the evidence accumulated in adults, there is far less known about the health benefits of nut consumption in children. The majority of literature in this area has focused on nut allergies, rather than on the purported health benefits of nut consumption [24,25]. Studies quantifying nut consumption in children and adolescents are limited and few large-scale epidemiological studies have evaluated the relationship between nut intake and dietary quality [9]. Only a small portion of children in the NHANES dataset (1999–2004) were identified as nut consumers (2.1–2.6%) based on the criterion of eating at least 0.25 ounces (~7 g) of nuts per day. Interestingly, those who did consume nuts had intakes between 3.4 and 4.0 ounces (96–113 g) per day for children aged 2–11 and 12–18 years respectively [9]. Using data from NHANES 2009–2012, Rehm et al. [3] modelled the effect of replacing typical snacks with nuts on nutrient intake. Consuming a diet rich in whole, raw tree nuts, would lower a child’s intake of sodium and empty calories (high energy/low nutrients), and increase monounsaturated fatty acids (MUFA) and polyunsaturated fatty acids (PUFA). Whilst nuts are considered part of a traditional Mediterranean diet [26], adolescents in the HELENA study in Europe were found to have low nut consumption with only 1.4% of total fat being contributed from nuts and seeds suggesting that total nut consumption was low [27]. Other studies have grouped nuts with a range of snack foods including sweets, potato chips and popcorn [28] or have not reported nut intake patterns at all [29], making it difficult to get a clear estimate of nut consumption in children and adolescents in these studies. The intake of nuts in children may be limited by policies to limit nuts being consumed at school to avoid possible food sharing and allergy risks, given that peanut allergies especially are highly prevalent [30,31]. 

To date, no study has comprehensively evaluated the effects of nut intake on children’s health. Consequently, the aim of the present study was to conduct a systematic review of randomized control trials (RCTs) to evaluate the effect of nut consumption on health outcomes and diet quality in children. 

## 2. Materials and Methods 

### 2.1. Protocol and Registration 

The systematic review was registered in International Prospective Register of Systematic Reviews (PROSPERO; registration number CRD420201861151) and conducted in accordance with the PRISMA (Preferred Reporting Items for Systematic Reviews and Meta-Analyses) guidelines [32]. 

#### 2.1.1. Eligibility Criteria 

The eligibility criteria used in this review are structured in a PICOS (Population, Intervention, Comparator, Outcome, Study design) format and summarized in Table 1. 

#### 2.1.2. Types of Studies

Randomized control trials (human studies) that compared nut consumption against a placebo or control (no nut consumption) were included. Studies needed to report at least one health-related outcome measure when participants consumed nuts. 

#### 2.1.3. Types of Participants

Child participants aged < 18 years.

#### 2.1.4. Types of Intervention

All nut varieties (including ground nuts and tree nuts) and duration (acute and chronic studies) of nut intake were considered. Nuts which were whole, raw, crushed, and a paste (e.g., nut butter) were included in the review.

#### 2.1.5. Exclusion Criteria

Studies were excluded if participants consumed nut oils or nuts that were chocolate coated or were in a product with other ingredients (e.g., nut muesli bar). Studies referring to allergy were also excluded.

#### 2.1.6. Types of Outcome Measures

All measures of health were considered as outcome measures in the review. These included gut health, adiposity, diet quality and cardiometabolic health (specifically lipid profiles, blood pressure, vascular function, glucose, and insulin metabolism).

#### 2.1.7. Limitations

No date restrictions were applied when searching in databases; however, studies were only eligible if they were published in a peer reviewed journal as a full text English article. 

### 2.2. Databases and Search Strategy

A systematic search was conducted on 30 April 2020 using the electronic databases PubMed (1996), Scopus (2004), Web of Science (1997), Cochrane Library (1993) and EMBASE (1980). Relevant key terms were sourced from previous systematic reviews on the health benefits of nuts (Barbour et al., 2014). An academic librarian (University of South Australia) reviewed the search strategy prior to any database searches. The following terms were searched in all databases: (nut OR nuts OR almond OR pistachio OR hazelnut OR walnut OR cashew OR macadamia OR pecan OR peanut OR “corylus avellana” OR “prunus dulcis” OR “prunus amygdalus” OR “pistacia vera” OR pistacia OR juglans OR “anacardium occidentale” OR “carya illinoinensis” OR “arachis hypogaea” OR groundnut OR hickory OR goober OR fibert OR “Brazil nut” OR “pine nut” OR “tree nut”) AND (child OR children OR adolescent OR adolescence OR pediatric OR paediatric OR youth OR teen* OR infant) AND (“cardiovascular health” OR “blood pressure” OR “systolic blood pressure” OR “diastolic blood pressure” OR SBP OR DBP OR “arterial compliance” OR “endothelial function” OR lipid OR “lipid profile” OR LDL OR “low density lipoprotein” OR HDL OR “high density lipoprotein” OR triglyceride OR cholesterol OR “lipid regulation” OR triacylglycerol OR TAG OR TG OR glucose OR insulin OR “glucose regulation” OR “insulin sensitivity” OR “insulin resistance” OR “blood sugar” OR “fasting glucose” OR “gut health” OR microbiome OR microbiota OR “cardiometabolic health” OR “diet quality” OR adiposity OR “body mass index” OR BMI OR “weight circumference” OR “body fat percentage” OR obesity OR “body composition” OR “waist to hip ratio”). Boolean operators “OR” and “AND” were used to combine the terms in the literature search. The search strategy was adapted for each database. 

### 2.3. Study Selection 

All search results were imported in “Endnote X8” and duplicate studies were removed based on title and author (LCM). Remaining results were exported into Covidence online software and title and abstract screening was completed by all authors (LCM, AMC, AMH, SC). Full-text articles were then independently screened for eligibility by two investigators (LCM screened all articles; AMC, AMH, SC equally split the role of the 2nd investigator). Disagreements were resolved by a third investigator who had not screened that specific article. 

### 2.4. Data Extraction 

Extracted data included: study characteristics (country, study design, number of participants), participant characteristics (gender, mean age, mean body mass index (BMI) status, health status), exposure (type, duration and amount of nut and control food consumed) and outcome measures (cardiometabolic health, diet quality and gut health). 

### 2.5. Quality Assessment

The quality of each eligible study was assessed by two investigators (LCM and AMC) using the 3-category Jadad scoring system [33]. Quality was determined by assessing how well a study design met the five criteria; randomization (question one was randomization mentioned and question two was a description of the randomization process mentioned), blinding (question three was the study double blinded and question four was the method of double blinding mentioned) and fate of all participants (question five was drop outs and reasons for withdrawal of participants described). Studies received one point for every assessment criterion they met (maximum five points). Studies with a quality assessment score of 0–2 were considered of low quality and studies with a score of 3–5 were of high quality.

### 2.6. Data Analysis

A descriptive analysis and effect size (Cohen’s D) were completed for each study by one investigator (LCM) in an excel spreadsheet [34]. Data presented as standard error of mean (SEM) were converted to standard deviation (SD) using the following formula:

(1) SD=SEM×square root (n), where n is the number of participants.

All SD data were then converted to pooled SD using the following formula:

(2) $$Pooled\;SD=square\;root\;\left(\frac{{\left(intervention\;group\;SD\right)}^{2}+{\left(control\;group\;SD\right)}^{2}}{2}\right)$$

Cohen’s D effect size was later calculated using the following formula:

(3) $$\left(\frac{\left(\mathrm{mean}\;\mathrm{intervention}\;-\;\mathrm{mean}\;\mathrm{control}\right)}{Pooled\;SD}\right)$$

Due to a lack of consistency in the way data were reported, no meta-analysis was undertaken.

## 3. Results

### 3.1. Study Selection

**Figure 1:**
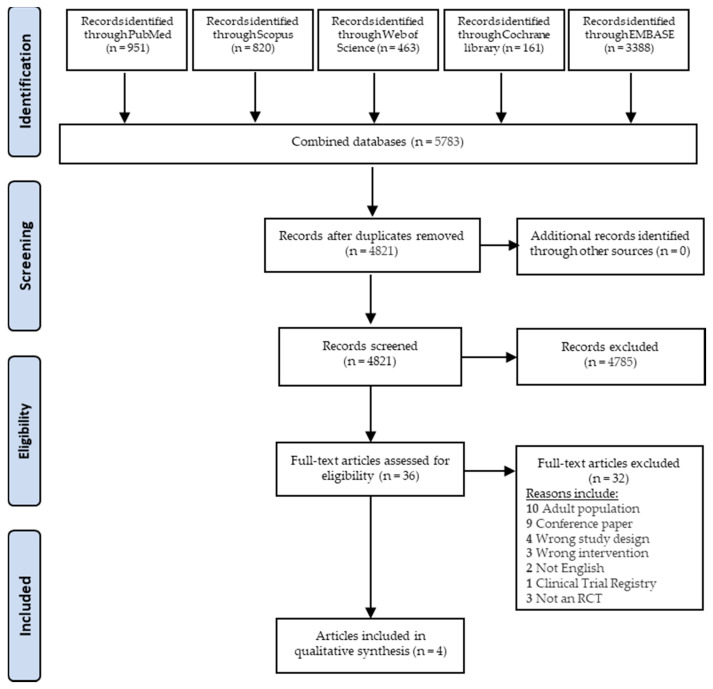
Flow diagram showing the screening process adapted from the Preferred Reporting Items for Systematic Reviews and Meta-Analyses (PRISMA) guidelines [32].

A total of 5783 articles were identified through database searching and imported for screening as shown in Figure 1. After removing duplicates, 4821 articles were screened by their title and abstract. This process removed 4784 articles resulting in 36 articles being screened via full text. A further 33 articles were excluded (as they mentioned allergy, reported no health outcome or tested nut oils, nuts that were chocolate coated or nuts in a product with other ingredients), resulting in four articles being eligible for inclusion in the review. In the four articles, different outcomes from one study were reported in two articles such that there were only three individual studies.

### 3.2. Quality Assesment

**Figure 2:**
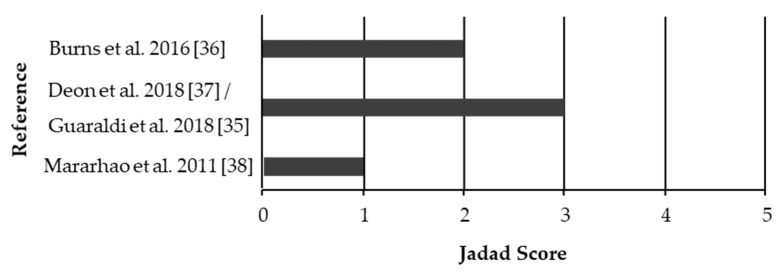
Quality assessment scores of eligible studies using the Jadad scoring system [33]. Articles were scored by two independent reviewers and no conflicts needed to be resolved.

The results of the quality assessment of eligible studies are shown in Figure 2. Scores ranged from 0 to 3 points; all articles received points for randomization [35,36,37,38] although only 1 study (outcomes reported in 2 articles) described the randomization process and participant withdrawal [35,37]. No studies were double blinded.

### 3.3. Study Characteristics

Characteristics of the 106 participants included in the three eligible studies are presented in Table 2 [35,36,37,38]. Deon et al. [37] and Guaraldi et al. [35] reported on different outcomes from the same study, therefore demographic data have been considered only once from these articles. The review included one study from Italy, one conducted in America and one conducted in Brazil. The number of participants included in each study varied; one included 60 participants, one study included 29 participants and one study included only 17 participants. Participants ranged in age from 4 to 15 years old. The majority of studies enrolled both males and females, except for Maranhao et al. [38] who included females only. More than half of the participants (60 out of 106) had known primary hyperlipidemia, all other participants were healthy.

Intervention duration varied throughout the studies and were either three [36], eight [35,37] or 16 weeks long [38]. Participants consumed almonds (whole almonds or the equivalent amount as almond butter) [36], hazelnuts (with or without skin) [35,37], or Brazil nuts [38]. Children ate raw almonds and Brazil nuts when these nut varieties were tested. The dose of nuts was relatively similar across studies and ranged from 15 to 30 g of nuts per day.

Different types of control interventions were implemented in the identified RCTs. All articles reported that control group participants were asked to avoid eating nuts. In addition to this, Deon et al. [37] Guaraldi et al. [35] provided the control group with dietary advice for hyperlipidemia, and Maranhao et al. [38] provided children in the comparator group with a daily placebo tablet containing lactose (the authors reported that lactose was chosen as it has little effect on children’s health outcomes).

Outcome measures varied between studies. Burns et al. [36] assessed diet quality using the Healthy Eating Index (HEI) tool, Deon et al. [37] reported on nutrient intake and assessed fatty acid composition of erythrocyte phospholipids as a biomarker of fatty acid intake, Burns et al. [36] reported on gastrointestinal health and Maranhao et al. [38] and Deon et al. [37] and Guaraldi et al. [35] measured various biomarkers of cardiometabolic health such as lipid profiles, glucose metabolism, inflammation, blood pressure, anthropometry, antioxidant capacity, oxidative stress and microcirculatory parameters. Markers of DNA damage were also assessed by Guaraldi et al. [35].

### 3.4. Dietary Profiles

The effect of almonds, hazelnuts and Brazil nuts on children’s diet profiles was explored in three articles [36,37,38] (Table 2). None of these studies reported significant changes in children’s total daily energy intake or fiber. Dietary intake was assessed using different methods in each study; 3 non-consecutive, unannounced 24-h dietary recalls (ASA24) were utilized by Burns et al. [36], weekly food diaries, before and after the intervention, with additional interviews during the study to monitor compliance were used by Deon et al. [37], while Maranhao et al. reported that the usual food intake was assessed by “dietary inquiry” before and after the study and participants were advised not to change their dietary habits [38]. Compliance with eating the nuts themselves was assessed through weekly diaries alone [36] or combined with also weighing returned portions of left over test foods [37] or through a combination of returned empty packets and serum selenium levels when Brazil nuts were provide as the test food [38]. Burns et al. [36] reported the average consumption in children per day (15 ± 0.57 g; intervention dose provided = 15 g) but it was unclear if compliance at an individual level was included in the statistical analyses. Deon et al. [37] reported that “hazelnut consumption was appreciated and well tolerated by all patients” but percent compliance or average amount consumed was not reported. When serum selenium levels were measured post intervention with Brazil nuts, there was a significant increase over time at the group level in those who consumed Brazil nuts but at the end of the intervention there was no difference between the Brazil nut or placebo groups [38].

Only one article reported that nuts, specifically hazelnuts, significantly increased fat intake; Deon et al. [37] reported children with primary hyperlipidemia who consumed 15–30 g of hazelnuts with and without skin for eight weeks significantly increased their intake of total fat and healthy fats such as MUFA, PUFA, Omega-3 PUFA, Omega-6 PUFA compared with baseline levels. Hazelnuts had no effect on children’s saturated fatty acids (SFA) and cholesterol intake or the serum lipid profile and fatty acid composition of erythrocyte membranes.

Deon et al. [37] reported that hazelnut consumption significantly decreased children’s carbohydrate intake but had no effect on protein intake compared to baseline. Conversely, while almond intake did not impact on carbohydrate intake it significantly increased children’s intake of protein during intervention compared to children who did not consume almonds [36]. Furthermore, Burns et al. [36] reported a reduction in empty calories (high energy/low nutrients) and an increase in magnesium, vitamin E and plant protein. Interestingly, while almonds had no significant effect on the intake of fruit, whole vegetables, greens and beans, whole grain, dairy, refined grains, total fat, MUFA, fiber, potassium, vitamin C and sodium compared to the control group, the changes in diet following almond consumption were sufficient to increase total HEI score compared to the control group (no almond intake) [36].

### 3.5. Cardiometabolic Health

The effect of almonds and hazelnuts on children’s cardiometabolic health was explored in three articles by Deon et al. [37], Guaraldi et al. [35] and Maranhao et al. [38] (Table 2). Inclusion of hazelnuts or Brazil nuts had no effect on children’s body weight or BMI [37,38]. Brazil nuts also had no effect on waist circumference, glucose or insulin metabolism (fasting glucose, insulin, Homeostatic Model of Assessment (HOMA)), inflammation (C-Reactive Protein) and HDL [38]. However, Maranhao et al. [38] found that there were reductions in triglycerides, total cholesterol, LDL and oxidized LDL compared to the control group who consumed a lactose containing placebo tablet.

Hazelnut consumption (15–30 g either with and without skins for eight weeks) by children with primary hyperlipidemia, had no effect on children’s levels of oxidized LDL, ratio of oxidized LDL to LDL, the ratio of oxidized LDL to HDL, or blood pressure. [35,37].

Maranhao et al. [38] reported that children who consumed 15–25 g of Brazil nuts for 16 weeks had an increased intake of the antioxidant mineral selenium. They failed to find any changes in biomarkers with antioxidant capacity but did reported a significant reduction in oxidized LDL. Guaraldi et al. [35] found a reduced amount of DNA damage in children who ate hazelnuts but no change in DNA strand breaks or oxidatively induced DNA damage compared with the control group [35].

Microvascular reactivity was significantly improved with the consumption of Brazil nuts as determined by a significant increase in the speed of red blood cells moving through the capillary at rest (compared to pre Brazil nut intake) and the speed of red blood cells passing through the capillary after 1-min arterial occlusion compared to no nut intake (16 weeks) [38]. However, Brazil nuts had no effect on biomarkers of antioxidant capacity (glutathione peroxidase 3 (GPX-3 and 8-epi-PGF2a), microcirculatory parameters (functional capillary density, TRBCV (time to reach max red blood cell velocity), afferent diameter, apical diameter and efferent diameter [38].

### 3.6. Gastrointestinal Health

The effect of almonds and hazelnuts on children’s gastrointestinal health was explored in one RCT conducted by Burns et al. [36] (Table 2). Several different methods were used to test gastrointestinal health including; measuring the operational taxonomic units (OTU) of bacteria inside the gut and different scores were used to assess the diversity of bacteria in the gut, and symptoms associated with gut bacteria.

Almonds were shown to alter one symptom score measured in the Gastrointestinal System Rating Scale (GSRS) [36]. Children who consumed 15 g of whole raw almonds or the equivalent in almond butter for three weeks, had reduced symptoms of constipation as their final GSRS constipation score reduced after three weeks compared to the control group (no almond intake) [36]. Almonds had no effect on other GSRS scores, including symptoms of diarrhea, abdominal pain, indigestion and reflux. Burns et al. [36] also discovered that almonds significantly altered the prevalence of various beneficial bacterial signatures at the genus and species level in children who consumed almonds at baseline compared to the end of the study (at three weeks). However, compared to children who were in the no almond control group, children who consumed almonds had no change in microbiota diversity (no changes in Shannon diversity index and the inverse Simpson diversity index), or the quantiles of *Bifidobacteria* spp and lactic acid bacteria.

## 4. Discussion

This is the first systematic review investigating the health benefits associated with nut consumption in children. This review included four articles reporting on outcomes from three RCTs with 106 participants (aged 18 years or younger). Over half of the data came from participants with primary hyperlipidemia. Children consumed either Brazil nuts, hazelnuts or almonds over a range of 3–16 weeks. A comprehensive range of health benefits were evaluated, broadly falling into the domains of diet quality, cardiometabolic health and gastrointestinal health.

### 4.1. Diet Quality

The inclusion of nuts in the diet of children has been shown to improve multiple dietary components, which is in agreement with previous observational studies that have found associations between consumption of nuts and higher diet quality in adults and children [3,9,10,12,39]. The protein and fiber content of nuts aids with satiety, which in turn may provide a mechanism for improving children’s diet quality through reducing the number of empty calories consumed [40]. Furthermore, nuts are rich in monounsaturated fat and essential vitamins and nutrients, which is healthier than many alternative snack foods that are high in SFA, added sugar and added salt [41].

### 4.2. Cardiometabolic Health

Several previous observational studies have linked nut consumption with cardiometabolic health benefits [9,12,42,43,44]. Children who eat nuts have lower total cholesterol and better endothelial function [9,12,44]. When Brazil nuts (15–25 g) were provided to healthy children for 16 weeks, there were reductions in triglycerides, total and LDL cholesterol and oxidized LDL [38]. However, the RCTs in this review that included children with primary hyperlipidemia, failed to find significant improvements in lipids or blood pressure when hazelnuts were incorporated into their diets [35,37]. The effect on lipids in healthy children and those with familial hypercholesterolemia must be considered separately to ensure clarity of effects in both populations.

### 4.3. Gastrointestinal Health

The findings from the current review on gastrointestinal outcomes found modest improvements in gut health with some changes in the prevalence of bacterial species at the species and genus levels and a reduction in constipation with almonds, but no improvement in microbial diversity. These results are less supportive than observational studies that have found an association between children eating a diet rich in nuts and increases in health-promoting bacteria and decreases in harmful bacteria [45]. Nuts contain components that are nondigestible to humans (for example polyphenols and polysaccharides), which are able to act as prebiotics and provide substrates for gut microbiota [46]. Studies in adults with walnuts [47,48], almonds [49], and pistachios [50] have shown that consumption over 3–16 weeks improves probiotic and butyric acid-producing species. It is important to expand our understanding of amounts that need to be consumed and length of supplementation to achieve benefits for the gut microbiota and to identify whether benefits are only afforded to certain populations.

### 4.4. Limitations and Future Studies

It is difficult to draw firm conclusions from this review due to the low number of studies investigating the effect of nut consumption on children’s health, the difference in quantity of nuts provided and the duration of interventions. Compared to the studies in adults, these nut volumes were considerably lower and may explain why changes in lipids in healthy children, and also those with primary hyperlipidemia, did not improve consistently in the studies included in this review. Further studies should investigate how different types of tree nuts and ground nuts, the dose of nuts, the form of nuts (e.g., whole, crushed, roasted, raw) and the length of nut intervention impacts children’s health outcomes. The quality of studies was mixed, which was driven by the lack of double blinding, which is common in whole food interventions, but also due to a lack of information describing the randomization process and participant withdrawal. Future RCTs should carefully follow the CONSORT reporting guidelines [51]. The studies did not report individual compliance with nut consumption clearly and future studies should also consider objective biomarkers of compliance where possible (such as serum selenium in the example where Brazil nuts were consumed).

## 5. Conclusions

Tree nuts and ground nuts are a nutrient-rich food shown in past observational studies to be associated with improved health in adults and children. The current systematic review of RCTs reported on a broad range of health outcomes but the paucity of data and lack of consistency limit the conclusions. It is clear that nuts are a healthy snack and should be recommended as part of a healthy diet for children; however, the current limited evidence indicates that they do not provide additional health benefits in hyperlipidemic children. Nevertheless, the replacement of energy-dense, nutrient-poor snacks with nuts has the potential to build healthy dietary habits as children develop into adults. Future interventions should focus on strategies to increase nut intake away from the school environment where restrictions due to allergy risk are not required.

## Figures and Tables

**Table 1:** Description of the PICOS criteria used to define the research question.

Parameter	Description
P-Population	Humans, children ≤ 18 years old
I-Intervention/variable of interest	Nut intake (whole, chopped, pastes)
C-Comparator	No nut intake, Received dietary advice, Placebo
O-Outcome	Cardiometabolic Health, Diet Quality, Adiposity and Gut Health
S-Study design	Randomized controlled trials

**Table 2:** Children’s health outcomes from nut consumption.

First Author (Year) Country	Number (N) and Characteristics of Participants	Study Design, (Length of Intervention)	Intervention Group(s)	Control Group	Health Outcomes from Nut Consumption
Burns et al. (2016) [36] USA	N = 29, Healthy males and females (15/14) 4 ± 0.2 years	Randomized, 2-phase crossover study (Each phase was 3 weeks with a 6-week wash-out)	15 g of almonds (whole, skin on) or the equivalent amount in almond butter per day	No almonds or products containing almonds	*Almond group compared to control group after intervention (3 weeks):* ↓ GSRS: constipation score, (*d* = −1.11) *Almond group at final week compared to baseline:* Significant changes in the prevalence of various bacterial signatures at the genus and species level, * *Almond group compared to control group during intervention (throughout 3 weeks):* ↑ HEI component score for total protein foods, (*d* = 1.01) ↑ HEI component score for fatty acids, (*d* = 1.0) ↑ HEI component score for seafood and plant proteins, (*d* = 2.47) ↑ HEI component score for empty calories (high energy/low nutrients), (*d* = 0.62) ↑ Total HEI Score, (*d* = 0.84) No change in HEI component score for total fruit, total vegetables, greens and beans, dairy, refined grain, sodium, whole fruit, whole grain. No change in mean energy intake or nutrient intake for protein, fat, monounsaturated fat, total fiber, potassium, energy, carbohydrate, sodium, vitamin C No changes in the microbiota profile (quantiles of Bifidobacteria spp and lactic acid bacteria, microbiota diversity measures (Shannon diversity index, inverse Simpson diversity index) and GSRS (Diarrhea, Abdominal pain, Indigestion, Reflux)
Deon et al. (2018) [37] Italy	N = 60, Hyperlipemic males and females (34/26) 11.6 ± 2.6 years	Randomized, single blind, three-arm, parallel- study (8 weeks)	Group 1: hazelnuts with skin (HZN + S), roasted 15–30 g/day, based on body weight Group 2-hazelnuts without skin (HZN-S), roasted 15–30 g/day, based on body weight	Group 3-dietary advice for hyperlipidemia (do not include nuts in diet)	*Intake of hazelnuts with and without skin at week 8 compared to baseline: * ↑ total Fat% of energy, (*d* = HZN + S: 1.13, HZN-S: 0.64) ↑ MUFA% of energy, (*d* = HZN + S: 0.45, HZN-S: 1.35) ↑ PUFA% of energy, (*d* = HZN + S: 1.82, HZN-S: 1.4) ↑ Omega 3% of energy, (*d* = HZN + S: 1.63, HZN-S: 0.8) ↑ Omega 6% of energy, (*d* = HZN + S: 1.95, HZN-S: 1.59) ↓ Carbohydrate intake% of energy, (*d* = HZN + S: −0.91, HZN-S: −0.42) No change in blood pressure, BMI, weight, daily energy and nutrient intakes (protein, SFA, fiber and cholesterol) No change in serum lipid profile and fatty acid composition of erythrocyte membranes except for: ↑ total MUFA composition of erythrocytes%, (*d* = HZN + S: 0.48, HZN-S: 0.57) ↑ the ratio of MUFA/PUFA composition of erythrocytes%, (*d* = HZN + S: 0.57, HZN-S: 0.67)
Guaraldi et al. (2018) [35] Italy	N = 60, Hyperlipidemic males and females, (34/26) 11.6 ± 2.6 years	Randomized, single blind, three-arm, parallel study (8 weeks)	Group 1- raw hazelnuts with skin (HZN + S) 15–30 g/day Group 2- hazelnuts without skin (HZN-S) 15–30 g/day	Group 3- dietary advice for hyperlipidemia (do not include nuts in diet)	*Intake of hazelnuts with and without skin: * ↓ levels of endogenous DNA damage (FGP-sensitive sites (% DNA in tail), (*d* = HZN + S: −1.13, HZN-S: −1.15) No change in level of DNA strand breaks and oxidatively induced DNA damage No change in levels of ox-LDL, ox-LDL/LDL and ox-LDL/HDL ratio
Maranhao et al. (2011) [38] Brazil	N = 17 Obese females 15.4 ± 2.0 years	Randomized, non-blinded, two-arm parallel study (16 weeks)	Brazil nuts, 15–25 g/day	Do not eat nuts and consume one capsule (placebo tablet) per day containing lactose	*Intake of Brazil nuts after 16 weeks compared to control:* ↓ triglycerides (mg/dL), * ↓ total cholesterol (mg/dL), * ↓ concentration of LDL (mg/dL), * No change in biomarkers with antioxidant capacity (GPX-3 and 8-epi-PGF2a) except for: *Intake of Brazil nuts after 16 weeks compared to baseline:* ↑ selenium (μg/L) (anti-inflammatory nutrient), * ↓ oxidized LDL (ng/mL), * ↑ RBCV max (mm/s) (Improved microvascular reactivity (compared to baseline and compared to control), * No changes in Body mass, BMI, WC, fasting insulin, glucose, HOMA, CRP, HDL-c No change in microcirculatory parameters (functional capillary density, TRBCV max, Afferent diameter, Apical diameter, Efferent diameter)

N: number; ns: not stated, **↑**: significantly increased (*p* < 0.05); **↓**: significantly decreased (*p* < 0.05); *d*: Cohen’ D effect size; * = No effect size calculated as data presented in heatmap or data presented as mean (1st tertile-3rd tertile); BMI: body mass index; CRP: C- reactive protein; FGP sensitive site: Formamidopyrimidine-DNA glycosylase-sensitive sites; GSRS: Gastrointestinal System Rating Scale; GPX-3: Glutathione peroxidase 3; HDL-c: concentration of high density lipoprotein; HEI: Healthy Eating Index; HOMA: Homeostatic Model of Assessment; HZN + S: hazelnuts with skin left on; HZN-S: hazelnuts with skin left off; LDL: Low density lipoprotein; MUFA: Monounsaturated fatty acids; Net H_2_0_2_: net hydrogen peroxide; ox-LDL: oxidized LDL; ox-LDL/LDL: ratio of oxidized LDL to LDL; ox-LDL/HDL: ratio of oxidized LDL to HDL; PUFA: Polyunsaturated fatty acids; SFA: Saturated fatty acids; RBCV: red blood cell velocity; RBCV max: maximum red blood cell velocity; TBRBC: time to reach max red blood cell velocity; 8-epi-PGF2a: 8-epi-prostaglandin F2alpha; age presented as mean ± standard deviation.

## Data Availability

The data presented in this study are available upon request from the corresponding author.
